# Workplace mental health law: comparative perspectives

**DOI:** 10.1093/occmed/kqag053

**Published:** 2026-07-28

**Authors:** Takenori Mishiba

**Affiliations:** Faculty of Law, Kindai University, Higashi-Osaka City 577-0813, Japan; Visiting Professor in Occupational Medicine, Tohoku University Graduate School of Medicine, Sendai, Japan

**Dear Editor** 

I appreciate Marcus Wong’s careful reading of my book and accept responsibility for the two errors he identified. The references to ‘discretion’ in relation to the UK Management Standards and to ACAS as enforcing the Equality Act should not have appeared in that form. Those were errors in the English expression of the book, and I should have detected them more carefully before publication. The reviewer was justified in pointing them out [1,2].

That said, I respectfully disagree with the suggestion that the title *Workplace Mental Health Law* is misleading because the UK has no single statute bearing that name. In the book, ‘law’ was used in an inclusive, comparative sense, encompassing statutes, case law, administrative guidance, compensation mechanisms and related legal and policy frameworks. It was never intended to imply that each jurisdiction has a single freestanding enactment called ‘workplace mental health law’ [2].

My further reservation is that the review gave little attention to the book’s central substantive thesis and scholarly contribution. The book’s purpose was not merely a descriptive comparison. It argued that workplace mental health problems cannot be addressed adequately only through formal categories of risk and liability; they also require attention to the characteristics, capacities, values and compatibility of both workers and organizations, and to the coordination of multiple stakeholders. In addition, the book presented, compared and analysed in detail the legal systems and practical operation of seven countries in this field. That, in itself, was an important and, at the time, highly unusual scholarly attempt [2].

In that respect, the review seemed to assess the book predominantly through matters of terminology and isolated error, without sufficient engagement with its principal comparative and normative contribution. That underlying argument was evidently regarded as sufficiently original and substantial to warrant publication by Routledge in the first place. It was later stated more explicitly in my editorial on the legal regulation of psychological hazards at work and was subsequently discussed in public in the exchange with Professor LaMontagne and colleagues [2–4].

My point is not that the book should have been immune from criticism. Rather, a fair overall assessment should have addressed not only two textual mistakes but also the book’s central argument and its comparative contribution. In comparative scholarship written across languages and legal cultures, precision is essential; but so too is engagement with the author’s underlying contribution. At the same time, leading researchers in Japan and the UK specializing in occupational health and related areas of law have recognized the academic value of this book.
